# Correction: Identification of the *Pseudomonas aeruginosa* AgtR-CspC-RsaL pathway that controls Las quorum sensing in response to metabolic perturbation and *Staphylococcus aureus*

**DOI:** 10.1371/journal.ppat.1013944

**Published:** 2026-02-10

**Authors:** The PLOS Pathogens Staff

S9 and S10 Figs were uploaded incorrectly. Please see the correct S9 and S10 Figs below. The publisher apologizes for the error.

## Supporting information

S9 Fig Competition between *S. aureus* and *P. aeruginosa* strains. Bacteria were grown overnight in LB at 37 °C. Indicated *P. aeruginosa* strains and *S. aureus* RN4220 were mixed at a 1:30 ratio and cocultured in LB at 37 °C for 14 h. The competitive indexes represent the ratio of *P. aeruginosa* to *S. aureus*. Data represent the mean ± standard deviation of the results from three samples. **, *P* < 0.01; *, *P* < 0.05 by Student’s *t* test.
(TIF)

S10 Fig The AgtR-CspC pathway is involved in sensing and competition against other bacteria. Bacteria were grown overnight in LB at 37 °C. (A) *P. aeruginosa* and *S. agalactiae* were mixed at a 1:3 ratio and cocultured in LB at 37 °C for 6 h. The competitive indexes represent the ratio of *P. aeruginosa* to *S. agalactiae*. (B) *P. aeruginosa* and *S. epidermidis* were mixed at a 1:30 ratio and cocultured in LB at 37 °C for 6 h. The competitive indexes represent the ratio of *P. aeruginosa* to *S. epidermidis*. Data represent the mean ± standard deviation of the results from three samples. ***, *P* < 0.001; **, *P* < 0.01; *, *P* < 0.05 by Student’s *t* test.
(TIF)
