# Supplementary figures and images for: Identification of the Pseudomonas aeruginosa AgtR-CspC-RsaL pathway that controls Las quorum sensing in response to metabolic perturbation and Staphylococcus aureus
Source: PLoS Pathog. 2025 Apr 8;21(4):e1013054. doi: 10.1371/journal.ppat.1013054 (PMC12051497; doi:10.1371/journal.ppat.1013054)

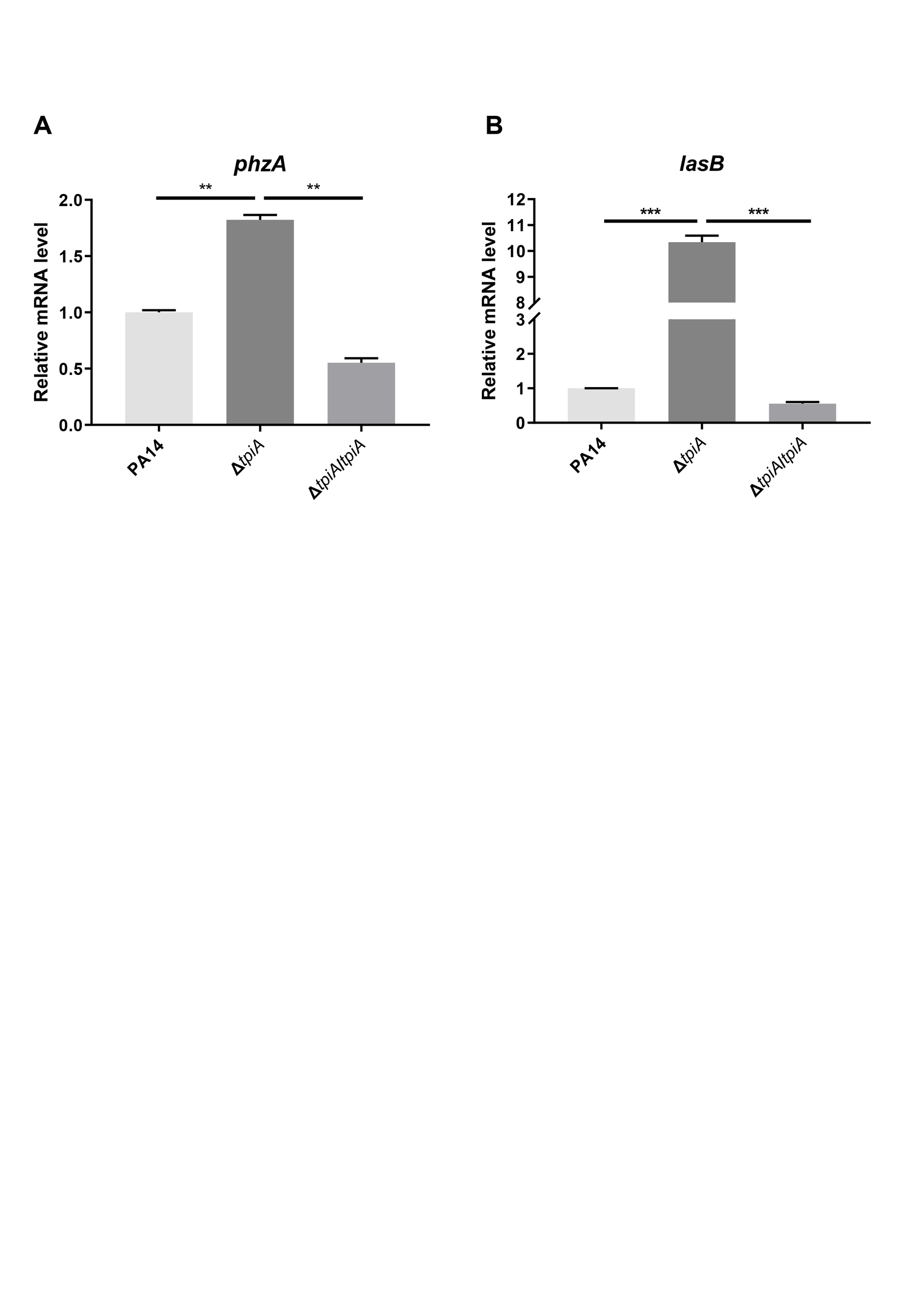

Supplement: S1 Fig — Bacteria were cultured in LB at 37 °C till OD600 reached 2.5. mRNA levels of phzA (A) and lasB (B) were determined by RT-qPCR. Data represent the mean ± standard deviation of the results from three samples. ***, P < 0.001; **, P < 0.01 by Student’s t test. (TIF) [file ppat.1013054.s001.tif]

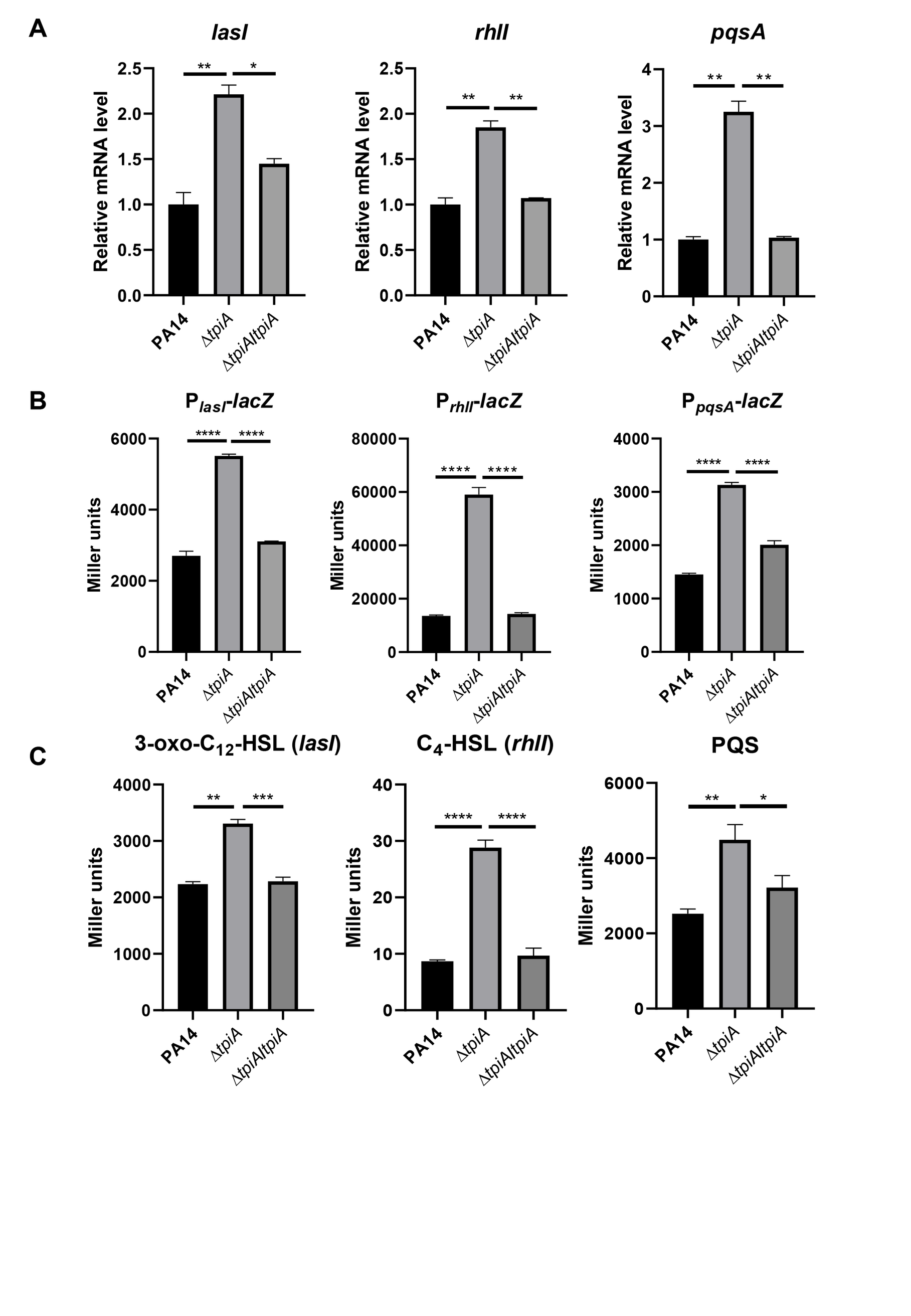

Supplement: S2 Fig — Bacteria were cultured in LB at 37 °C till OD600 reached 0.5. (A) mRNA levels of the QS signaling molecule synthesis genes, including lasI, rhlI and pqsA were determined by RT-qPCR. (B) Promoter activities of lasI, rhlI and pqsA were detected by β-galactosidase activity assay. (C) 3-Oxo-C12-HSL, C4-HSL and the PQS signal molecules (PQS and HHQ) levels in the supernatants of indicated strains were measured with corresponding reporter strains using β-galactosidase activity assay. Data represent the mean ± standard deviation of the results from three samples. ****, P < 0.0001; ***, P < 0.001; **, P < 0.01; *, P < 0.05 by Student’s t test. (TIF) [file ppat.1013054.s002.tif]

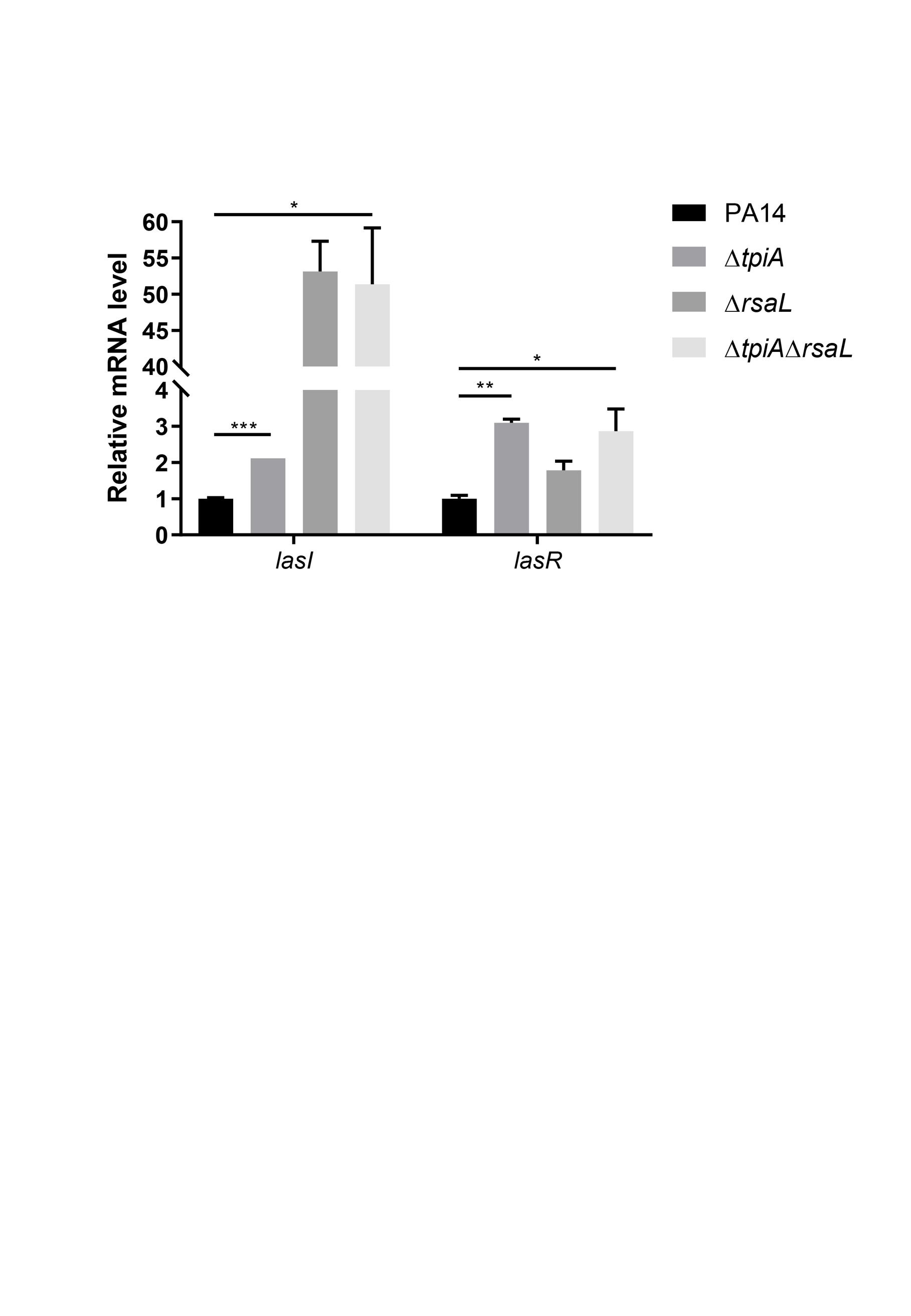

Supplement: S3 Fig — Bacteria were cultured in LB at 37 °C till OD600 reached 2.5. mRNA levels of lasI and lasR were determined by RT-qPCR. Data represent the mean ± standard deviation of the results from three samples. ***, P < 0.001; **, P < 0.01; *, P < 0.05 by Student’s t test. (TIF) [file ppat.1013054.s003.tif]

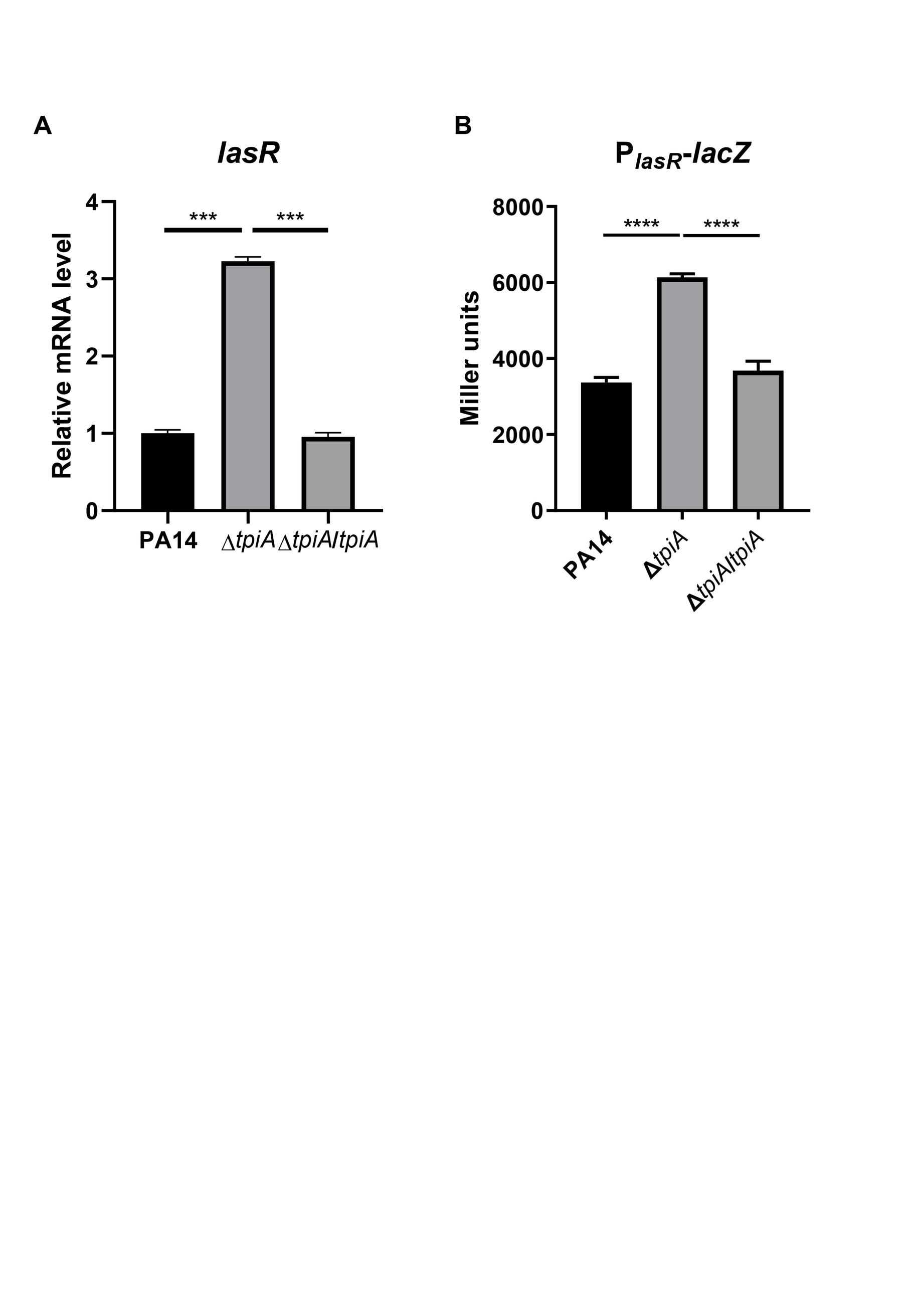

Supplement: S4 Fig — Bacteria were cultured in LB at 37 °C till OD600 reached 2.5. (A) mRNA levels of lasR were determined by RT-qPCR. (B) Promoter activities of lasR were determined by β-galactosidase activity assay. Data represent the mean ± standard deviation of the results from three samples. ****, P < 0.0001; ***, P < 0.001 by Student’s t test. (TIF) [file ppat.1013054.s004.tif]

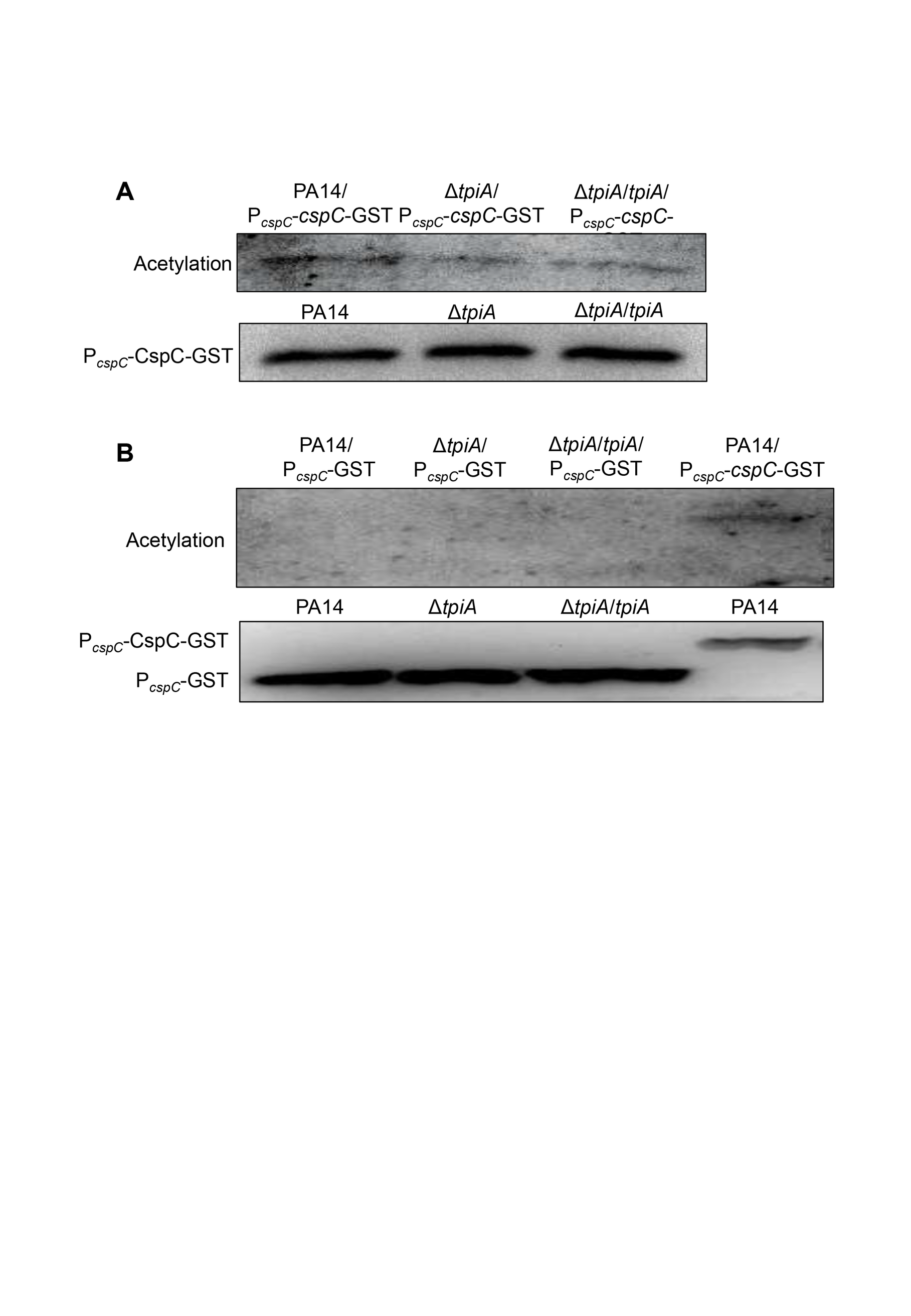

Supplement: S5 Fig — Bacteria containing cspC-gst or gst driven by PcspC were cultured in LB at 37 °C till OD600 reached 2.5. Acetylation and total amounts of the purified CspC-GST (A) and GST (B) were determined by Western Blot. (TIF) [file ppat.1013054.s005.tif]

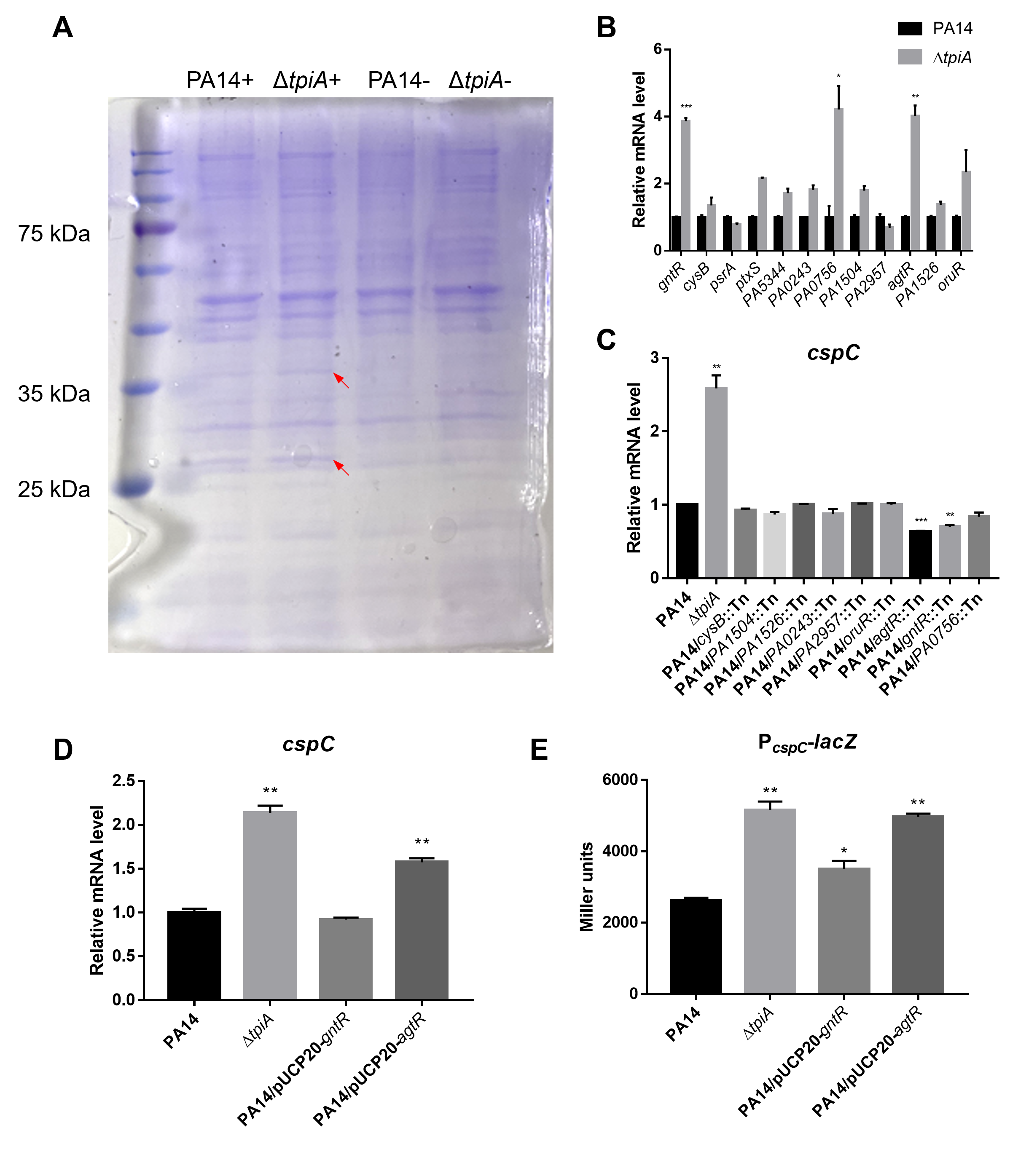

Supplement: S6 Fig — (A) Identification of candidate proteins binding to the PcspC promoter. The biotin-labeled PcspC promoter fragment was incubated with the cell lysate of PA14 or the ΔtpiA mutant, followed by purification with streptavidin-conjugated beads. The protein bands different from the control samples (cell lysates without DNA fragment) are indicated with arrows. (B) mRNA levels of the candidate regulatory genes were determined by RT-qPCR. (C) mRNA levels of cspC in transposon insertion mutants of the candidate genes were determined by RT-qPCR. (D) mRNA levels of cspC in indicated strains were determined by RT-qPCR. (E) Promoter activities of cspC were detected by β-galactosidase activity assay. Data represent the mean ± standard deviation of the results from three samples. ***, P < 0.001; **, P < 0.01; *, P < 0.05 by Student’s t test. (TIF) [file ppat.1013054.s006.tif]

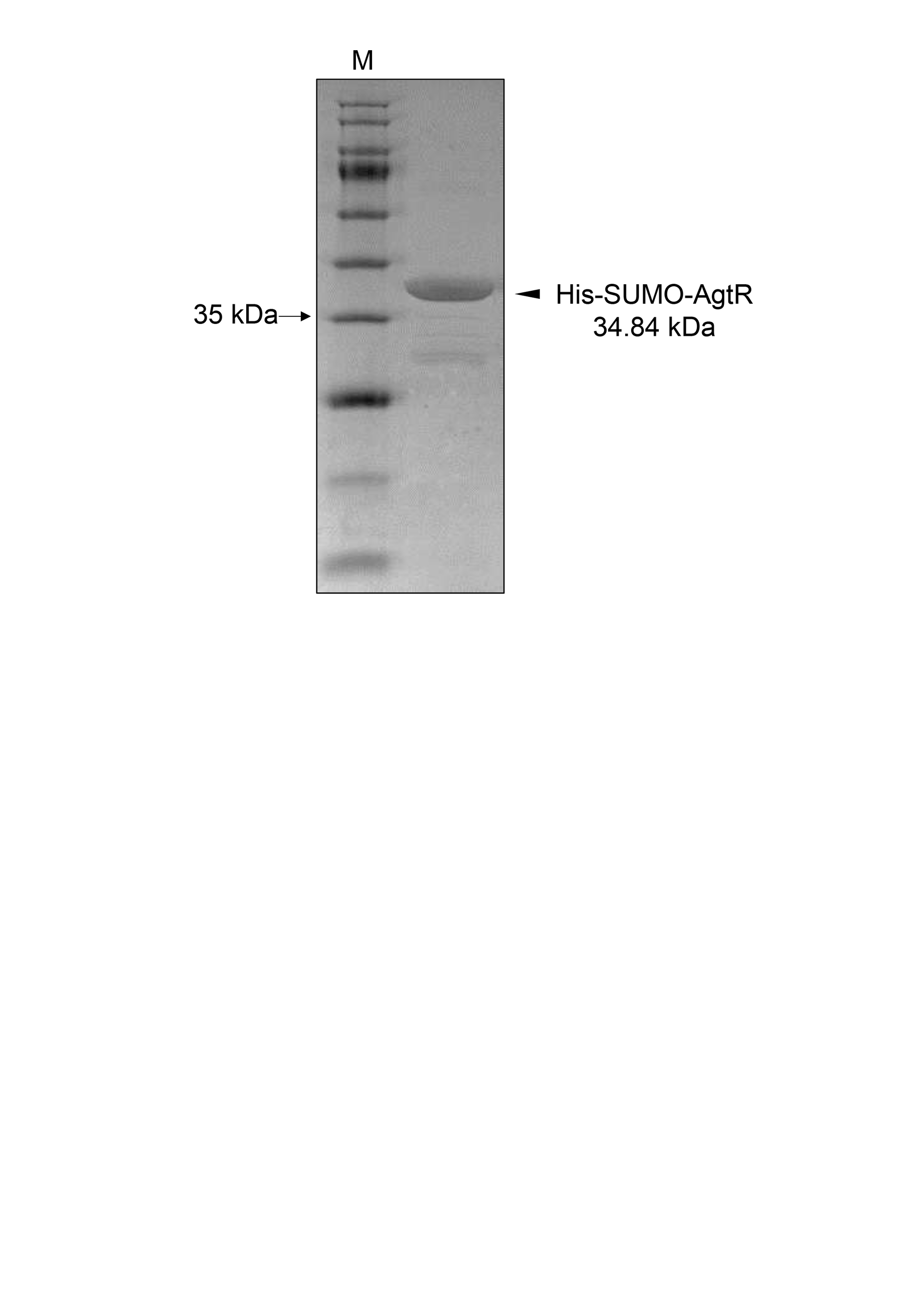

Supplement: S7 Fig — AgtR fused with a N-terminus 6×His tag and a solubilizing tag SUMO was purified with Ni-affinity chromatography and subjected to SDS-PAGE analysis and Coomassie blue staining. The purified protein is indicated by an arrowhead. M, protein marker. (TIF) [file ppat.1013054.s007.tif]

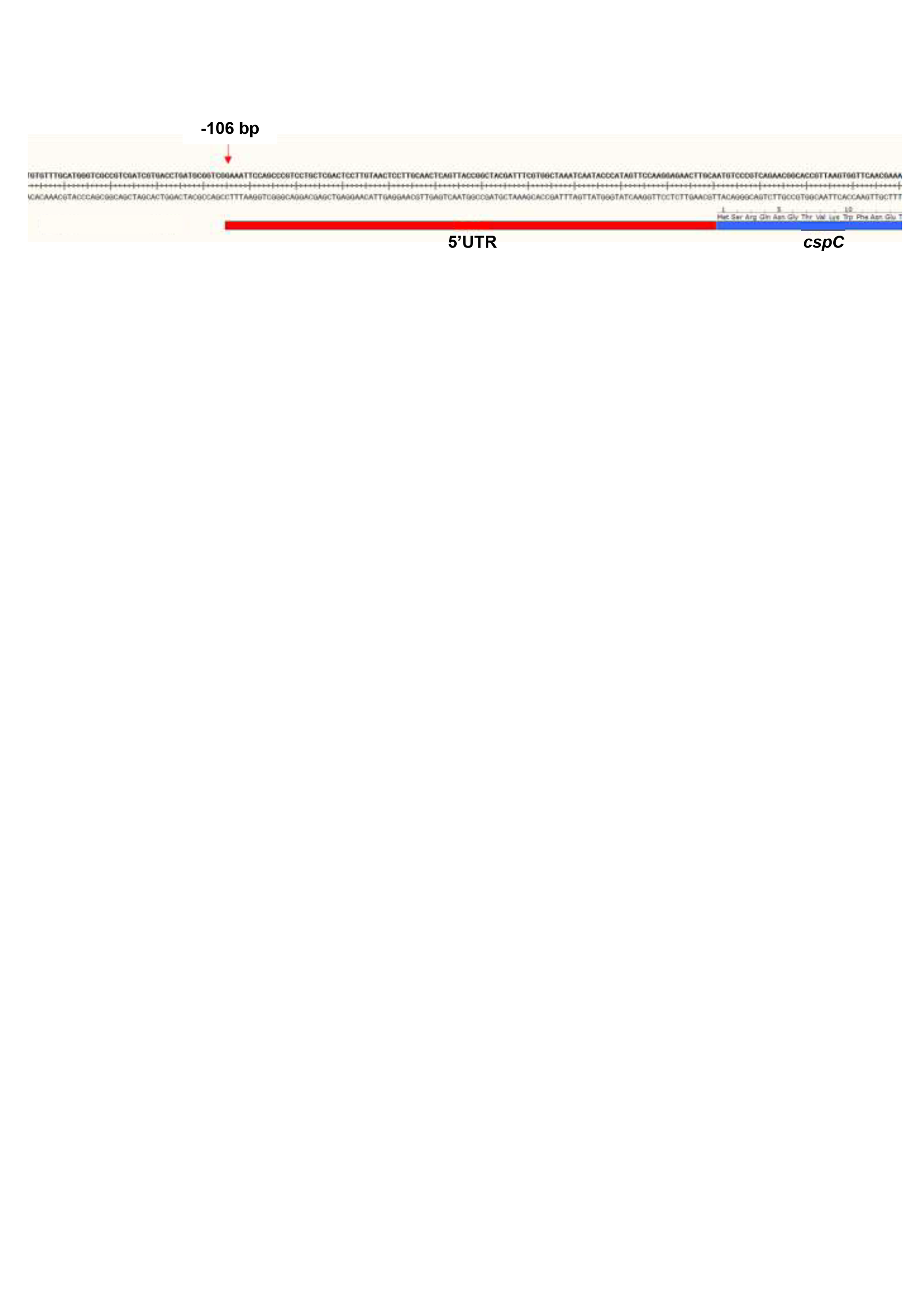

Supplement: S8 Fig — The transcriptional start site was determined by 5’-RACE assay and indicated by a red arrow. (TIF) [file ppat.1013054.s008.tif]
