# Supplementary material for: Identification of the Pseudomonas aeruginosa AgtR-CspC-RsaL pathway that controls Las quorum sensing in response to metabolic perturbation and Staphylococcus aureus
Source: PLoS Pathog. 2025 Apr 8;21(4):e1013054. doi: 10.1371/journal.ppat.1013054 (PMC12051497; doi:10.1371/journal.ppat.1013054)
Supplement: S1 Table — (DOCX) [file ppat.1013054.s011.docx]

**S1 Table. Culture time and concentrations of the bacteria at the corresponding OD_600_ (three samples of each strain).**

| **Strain** | **PA14** | **Δ*tpiA*** | **Δ*tpiA*/*tpiA*** |
| --- | --- | --- | --- |
| Culture time*  (OD_600_ reached 0.5) | 2 h | 2 h 20 min | 2 h |
| CFU/mL (OD_600_ at 0.5) | 2.70×10^8^ | 3.76×10^8^ | 3.88×10^8^ |
|  | 3.29×10^8^ | 3.53×10^8^ | 3.06×10^8^ |
|  | 2.24×10^8^ | 3.76×10^8^ | 2.35×10^8^ |
| Culture time*  (OD_600_ reached 2.5) | 4 h 45 min | 8 h 30 min | 4 h 45 min |
| CFU/mL (OD_600_ at 2.5) | 2.80×10^9^ | 3.40×10^9^ | 3.20×10^9^ |
|  | 3.00×10^9^ | 3.10×10^9^ | 3.00×10^9^ |
|  | 3.00×10^9^ | 3.10×10^9^ | 3.30×10^9^ |

*****, around 8×10^7^ CFU/mL bacteria were inoculated in LB and grown at 37 ºC with agitation at 200 rpm.
