# Supplementary material for: Identification of the Pseudomonas aeruginosa AgtR-CspC-RsaL pathway that controls Las quorum sensing in response to metabolic perturbation and Staphylococcus aureus
Source: PLoS Pathog. 2025 Apr 8;21(4):e1013054. doi: 10.1371/journal.ppat.1013054 (PMC12051497; doi:10.1371/journal.ppat.1013054)
Supplement: S2 Table — (DOCX) [file ppat.1013054.s012.docx]

**S2 Table. Primers used in this study.**

| **Primer** | **Sequence (5’→3’)** | **Purpose** |
| --- | --- | --- |
| rpsL-qPCR-F | TATGCCGTGTACGTCTGA | qPCR |
| rpsL-qPCR-R | CACTACGCTGTGCTCTTG | qPCR |
| lasI-qPCR-F | CTGGGCGAGATGCACAAGT | qPCR |
| lasI-qPCR-R | AGCAACCGAAAACCTGGG | qPCR |
| lasR-qPCR-F | CGAGCGACCTTGGATTC | qPCR |
| lasR-qPCR-R | GTAGTTGCCGACGATGAA | qPCR |
| rhlI-qPCR-F | CGAATTGCTCTCTGAATC | qPCR |
| rhlI-qPCR-R | TTCTCGATGAAGACTTGA | qPCR |
| pqsA-qPCR-F | CATGTGTTCCTCGCCAACCG | qPCR |
| pqsA-qPCR-R | GTGTGTCCTTCGCGGTCCAC | qPCR |
| phzA-qPCR-F | CCACTACATCCATTCCTTC | qPCR |
| phzA-qPCR-R | AATTTCTGCATCGGGTTC | qPCR |
| lasB-qPCR-F | GACCGAGAATGACAAAGTGGAAC | qPCR |
| lasB-qPCR-R | CGGGAATCAGGTAGGAGACGT | qPCR |
| rsaL-qPCR-F | ACGAGAGAACACAGCCCCA | qPCR |
| rsaL-qPCR-R | AGAAATGCAAAAGCAGATATATAGG | qPCR |
| rsaL-GST-qPCR-F | GCAAATTACCGATCGCCAGCTC | qPCR |
| GST-qPCR-R | GTTGCACAAGGCCCTTAATTTTCC | qPCR |
| cspC-qPCR-F | TTCAACGAAACCAAAGGCTA | qPCR |
| cspC-qPCR-R | GATGGCACGGAAGTGAAC | qPCR |
| agtR-qPCR-F | AGCAACTGATTGGCATGGCC | qPCR |
| agtR-qPCR-R | AGGGTTTCCAGCAACTGCTCG | qPCR |
| PA1504-qPCR-F | ATCACCTGCTCTTCGCCATC | qPCR |
| PA1504-qPCR-R | ACGATGGAAATCTGCCAATCG | qPCR |
| PA0756-qPCR-F | TGATGAGCAAGGAGCAACTGG | qPCR |
| PA0756-qPCR-R | AGGCGGTGGACGTAGATCTC | qPCR |
| PA2957-qPCR-F | ATTCCGAAGACGTAGCCCGC | qPCR |
| PA2957-qPCR-R | TCGAACAGGCGGTAGCGCTG | qPCR |
| PA1526-qPCR-F | AACATGTGGAAAGCCTCTACGC | qPCR |
| PA1526-qPCR-R | AGGAACGGTGCCAGCTCG | qPCR |
| PA0243-qPCR-F | ACGCATACCTCGAAGCGGATG | qPCR |
| PA0243-qPCR-R | TGTAGAGCTTCTCCAGCACGG | qPCR |
| psrA-qPCR-F | ACCTGTCGATCTTCATGCGC | qPCR |
| psrA-qPCR-R | ACCTTGCCGTAGACCTCTTCC | qPCR |
| PA5344-qPCR-F | ATCTACACCATCGGTCCCTAC | qPCR |
| PA5344-qPCR-R | AAGTTCTCCTCGATGTACAGCG | qPCR |
| cysB-qPCR-F | ACCTGATCATGATGCCCTGC | qPCR |
| cysB-qPCR-R | TCAGTTTCGGCAGCTTGGTC | qPCR |
| gntR-qPCR-F | ACGAGGAAGAGGACCTGATCC | qPCR |
| gntR-qPCR-R | TCTCGGTGCGCTCGAAGC | qPCR |
| ptxS-qPCR-F | AGTCCTACAACGTCGAAGGAC | qPCR |
| ptxS-qPCR-R | ACCAGCACCATCGGCAGTTC | qPCR |
| oruR-qPCR-F | ACTTCCCCGATCTCGAACAGG | qPCR |
| oruR-qPCR-R | ACCTGCTGGTAGGTGGTGC | qPCR |
| EcoRI-PrsaL-F | CCGGAATTCTAACTTCACTTCCTCCAAATAGGAAGCTG | *rsaL* promoter cloning with pDN19lacΩ or fusion of *rsaL-gst* cloning on a promoterless pUCP20 |
| BamHI-hrsaL-R | CGCGGATCCCAGGTATATAGGGAAGGGCAGGTTC | *rsaL* promoter cloning with pDN19lacΩ |
| EcoRI-PcspC-F | CCGGAATTCTGGATCGGCGTCATCTCC | *cspC* promoter cloning with pDN19lacΩ |
| BamHI-PcspC-R | CGCGGATCCTGCAAGTTCTCCTTGGAACTATGGG | *cspC* promoter cloning with pDN19lacΩ |
| PcspC-F-Biotin | TGGATCGGCGTCATCTCC | DNA pull-down assay |
| FAM-PcspC-F | TTGCCGCGTGCGATCGC | DNase I footprint assay |
| PcspC-R | TGCAAGTTCTCCTTGGAACTATGG | DNA pull-down assay |
| PcspC-1-F | TGCAAGTTCTCCTTGGAACTATGG | EMSA |
| PcspC-2-F | TCTTTGGCCAGTACCGTTCGTC | EMSA |
| PcspC-3-F | GTTCGCTTTCCTCAGTTTTTTGTC | EMSA |
| PcspC-4-F | AGTGCTGACACTGTGTTTGC | EMSA |
| PcspC-5-F | GTTCGCTTTCCTCAGTTTTTTGTCGTAGAGTGCTGACAC | EMSA |
| PcspC-5-R | GTGTCAGCACTCTACGACAAAAAACTGAGGAAAGCGAAC | EMSA |
| mutPcspC-R | CACAGTGTCAGCTAAATACGACAAAAAACTGAGG | P*cspC* mutation |
| mutPcspC-F | CCTCAGTTTTTTGTCGTATTTAGCTGACACTGTG | P*cspC* mutation |
| BamHI-GST-R | CGCGGATCCTTTTGGAGGATGGTCGCCACC | fusion of *rsaL-gst* cloning on a promoterless pUCP20 |
| PrsaL-rsaL-overlap-R | CTCTCGTGTGAAGCCATTTATAAAATTATGAAATTTGCATAAATTCTTCAGCTTCC | fusion of *rsaL-gst* cloning on a promoterless pUCP20 |
| rsaL-overlap-F | GCAAATTTCATAATTTTATAAATGGCTTCACACGAGAGAACAC | fusion of *rsaL-gst* cloning on a promoterless pUCP20 |
| delta-cspC-F | CAGCGACAGGCAATGTATCTTTGG | *cspC* deletion |
| delta-cspC-R | CATCAGCCAGGACTGCTCGATC | *cspC* deletion |
| agtR-U-F | CCCAAGCTTTGGTCGGCTACAGCGCTCG | *agtR* deletion |
| agtR-U-R | AGCAAAACCTGGGCGAATCTTTGCTGCCTCACGCTGTTC | *agtR* deletion |
| agtR-D-F | AGGCAGCAAAGATTCGCCCAGGTTTTGCTCGC | *agtR* deletion |
| agtR-D-R | CGCGGATCCCCGTAGGCGATGTTCTCGGC | *agtR* deletion |
| phz1-U-F | CCTCCTCGGTGGTGCGG | *phz1* operon deletion |
| phz1-U-R | TGGGTACTTCTCGGGTTACGG | *phz1* operon deletion |
| phz1-D-F | CATCCGCCCCAGCCGA | *phz1* operon deletion |
| phz1-D-R | GCGTGATGCGTCCCCG | *phz1* operon deletion |
| EcoRI-agtR-F | CCGGAATTCGTGGTGATCCGAGTCCTGGTCG | *agtR* cloning on the plasmid pUCP20 |
| BamHI-agtR-R | CGCGGATCCTCAATGGTGATGGTGATGATGGAGCAGGCGGTGCTCCATC | *agtR* cloning on the plasmid pUCP20 |
| EcoRI-gntR-F | CCGGAATTCATGAGCATCACCAAGAACGACAAG | *gntR* cloning on the plasmid pUCP20 |
| BamHI-gntR-R | CGCGGATCCTCAATGGTGATGGTGATGATGGGTGCTCTCCCGCGCCATC | *gntR* cloning on the plasmid pUCP20 |
| EcoRI-AgtR-F | CCGGAATTCGTGATCCGAGTCCTGGTCGC | *agtR* cloning on the plasmid pET-His-SUMO |
| HindIII-AgtR-R | CCCAAGCTTTCAGAGCAGGCGGTGCTC | *agtR* cloning on the plasmid pET-His-SUMO |
| 5-AP | CGACTTGAACTCCACCCCCCCCCCC | 5’-RACE |
| 5-NP | CGACTTGAACTCCACC | 5’-RACE |
| cspC-RACE-R1 | GTTGATCACCTGAACGCGCT | 5’-RACE |
| cspC-RACE-R2 | GCACGGAAGTGAACGAAAACGT | 5’-RACE |
