# Supplementary material for: Identification of the Pseudomonas aeruginosa AgtR-CspC-RsaL pathway that controls Las quorum sensing in response to metabolic perturbation and Staphylococcus aureus
Source: PLoS Pathog. 2025 Apr 8;21(4):e1013054. doi: 10.1371/journal.ppat.1013054 (PMC12051497; doi:10.1371/journal.ppat.1013054)
Supplement: S3 Table — (DOCX) [file ppat.1013054.s013.docx]

**S3 Table**. **Plasmids and strains used in this study.**

| **Name** | **Description** | **Source (Reference)** |
| --- | --- | --- |
| **Plasmid** |  |  |
| P*_lasI_*-*lacZ* | pUCP20 lacks promoter with *lasI* promoter; Ap^r^ | [1] |
| P*_rhlI_*-*lacZ* | pUCP20 lacks promoter with *rhlI* promoter; Ap^r^ | [1] |
| P*_pqsA_*-*lacZ* | pDN19lacΩ with *pqsA* promoter; Ap^r^ | [2] |
| P*_lasR_*-*lacZ* | pUCP20 lacks promoter with *lasR* promoter; Ap^r^ | [1] |
| P*_rsaL_*-*lacZ* | pDN19lacΩ with *rsaL* promoter; Tc^r^ | This study |
| P*_cspC_*-*lacZ* | pDN19lacΩ with *cspC* promoter; Tc^r^ | This study |
| mutP*_cspC_*-*lacZ* | pDN19lacΩ with *cspC* mutant promoter; Tc^r^ | This study |
| pUCP20-P*_rsaL_*-*rsaL-*GST | *rsaL* promoter of PA14 fused to ­*rsaL*-GST on a promoterless pUCP20; Ap^r^ | This study |
| pMMB67EH-74-*rsaL*-GST | 74utr- *rsaL* -GST driven by an inducible tac promoter; Ap^r^ | [1] |
| pMMB67EH-37-*rsaL*-GST | 37utr- *rsaL* -GST driven by an inducible tac promoter; Ap^r^ | [1] |
| pMMB67EH-*rsaL*-GST | *rsaL* -GST driven by an inducible tac promoter; Ap^r^ | [1] |
| pUCP20-P*_cspC_*-GST | *cspC* promoter of PA14 fused to GST on a promoterless pUCP20; Ap^r^ | [1] |
| pUCP20-P*_cspC_*-*cspC*-GST | *cspC* promoter of PA14 fused to *cspC*-GST on a promoterless pUCP20; Ap^r^ | [1] |
| pEx18Tc-Δ*cspC* | *cspC* gene deletion suicide plasmid; Tc^r^ | [1] |
| pEx18Tc-Δ*rsaL* | *rsaL* gene deletion suicide plasmid; Tc^r^ | [1] |
| pEx18Tc-Δ*agtR* | *agtR* gene deletion suicide plasmid; Tc^r^ | This study |
| pEx18Tc-Δ*phz1* | *phzA1B1C1D1E1F1G1* operon deletion suicide plasmid; Tc^r^ | This study |
| pUCP20-*agtR* | *agtR* on the plasmid pUCP20, Ap^r^ | This study |
| pUCP20-*gntR* | *gntR* on the plasmid pUCP20, Ap^r^ | This study |
| pET-His-SUMO | Clone vector containing His and SUMO tags, Kan^r^ | [3] |
| pET-His-SUMO-*agtR* | *agtR* on the plamid pET-His-SUMO, Kan^r^ | This study |
| **Strain** |  |  |
| ***P. aeruginosa*** |  |  |
| PA14 | Wild type strain | [4] |
| Δ*tpiA* | PA14 *tpiA* gene deletion mutant | [5] |
| Δ*tpiA* / *tpiA* | Δ*tpiA* complementation with *tpiA* inserted on chromosome, Gm^r^ | [5] |
| Δ*rsaL* | PA14 rsaL gene deletion mutant | [1] |
| Δ*tpiA*Δ*rsaL* | PA14 *tpiA rsaL* dual-deletion mutant | This study |
| Δ*cspC* | PA14 *cspC* gene deletion mutant | [1] |
| Δ*tpiA*Δ*cspC* | PA14 *tpiA cspC* dual-deletion mutant | This study |
| Δ*pqsA* | PAO1 *pqsA* gene deletion mutant | [2] |
| Δ*agtR* | PA14 *agtR* gene deletion mutant | This study |
| Δ*tpiA*Δ*agtR* | PA14 *tpiA agtR* dual-deletion mutant | This study |
| Δ*phz1* | PA14 *phzA1B1C1D1E1F1G1* operon deletion mutant | This study |
| Δ*phz1*Δ*agtR* | PA14 deleted of *phzA1B1C1D1E1F1G1* operon and *agtR* gene | This study |
| PA14/*agtR*::Tn | PA14 *agtR* gene transposon insertion mutant | [6] |
| PA14/*gntR*::Tn | PA14 *gntR* gene transposon insertion mutant | [6] |
| PA14/*cysB*::Tn | PA14 *cysB* gene transposon insertion mutant | [6] |
| PA14/*psrA*::Tn | PA14 *psrA* gene transposon insertion mutant | [6] |
| PA14/*ptxS*::Tn | PA14 *ptxS* gene transposon insertion mutant | [6] |
| PA14/*PA5344*::Tn | PA14 *PA5344* gene transposon insertion mutant | [6] |
| PA14/*PA0243*::Tn | PA14 *PA0243* gene transposon insertion mutant | [6] |
| PA14/*PA0756*::Tn | PA14 *PA0756* gene transposon insertion mutant | [6] |
| PA14/*PA1504*::Tn | PA14 *PA1504* gene transposon insertion mutant | [6] |
| PA14/*PA2957*::Tn | PA14 *PA2957* gene transposon insertion mutant | [6] |
| PA14/*PA1526*::Tn | PA14 *PA1526* gene transposon insertion mutant | [6] |
| PA14/*oruR*::Tn | PA14 *oruR* gene transposon insertion mutant | [6] |
| ***E. coli*** |  |  |
| DH5α | F^-^, φ80d*lacZ*ΔM15, Δ(*lacZYA-argF*)U169, *deoR*, *recA1*, *endA1*, *hsdR17*(r_k_^-^,m_k_^+^), *phoA*, *supE44*, λ^-^, *thi-1*, *gyrA96*, *relA1* | TransGen |
| S17-1 | *recA*, *pro*, *hsdR*, RP4-2-Tc::Mu-Km::Tn7 | Stratagene |
| pECP64 | DH5α containing pECP64 plasmid, 3-oxo-C12-HSL reporter strain | [7] |
| pECP61.5 | DH5α containing pECP61.5 plasmid, C4-HSL reporter strain | [7] |
| BL21(DE3) | F–, *ompT*, *hsdS_B_* (r_B_–, m_B_–), *gal*, *dcm*(DE3) | Thermo Scientific |
| ***S. aureus*** |  |  |
| RN4220 | 8325-4, r-, initial recipient for modification of plasmids | [8] |
| ***S. agalactiae*** |  |  |
| COH1 | a highly encapsulated serotype Ⅲ clinical isolate with proven virulence in animal models of sepsis and meningitis | [9] |
| ***S. epidermidis*** | ATCC 35984 | [9] |

**References**

1. Li S, Weng Y, Li X, Yue Z, Chai Z, Zhang X, et al. Acetylation of the CspA family protein CspC controls the type III secretion system through translational regulation of *exsA* in *Pseudomonas aeruginosa*. Nucleic Acids Res. 2021; 49(12):6756-6770. https://doi: 10.1093/nar/gkab506. PMID: 34139014.
2. Pan X, Liang H, Zhao X, Zhang Q, Chen L, Yue Z, et al. Regulatory and structural mechanisms of PvrA-mediated regulation of the PQS quorum-sensing system and PHA biosynthesis in *Pseudomonas aeruginosa*. Nucleic Acids Res. 2023; 51(6):2691-2708. https://doi: 10.1093/nar/gkad059. PMID: 36744476.
3. Liang Q, Yan J, Zhang S, Yang N, Li M, Jin Y, et al. CtrA activates the expression of glutathione S-transferase conferring oxidative stress resistance to *Ehrlichia chaffeensis*. Front Cell Infect Microbiol. 2022; 12:1081614. https://doi: 10.3389/fcimb.2022.1081614. PMID: 36579340.
4. Liberati NT, Urbach JM, Miyata S, Lee DG, Drenkard E, Wu G, et al. An ordered, nonredundant library of *Pseudomonas aeruginosa* strain PA14 transposon insertion mutants. Proc Natl Acad Sci U S A. 2006; 103(8):2833-8. Epub 2006/02/13. Erratum in: Proc Natl Acad Sci U S A. 2006/12/26; 103(52):19931. https://doi: 10.1073/pnas.0511100103. PMID: 16477005.
5. Xia Y, Wang D, Pan X, Xia B, Weng Y, Long Y, et al. TpiA is a key metabolic enzyme that affects virulence and resistance to aminoglycoside antibiotics through CrcZ in *Pseudomonas aeruginosa*. mBio. 2020; 11(1):e02079-19. https://doi: 10.1128/mBio.02079-19. PMID: 31911486.
6. Liberati NT, Urbach JM, Miyata S, Lee DG, Drenkard E, Wu G, et al. An ordered, nonredundant library of *Pseudomonas aeruginosa* strain PA14 transposon insertion mutants. Proc Natl Acad Sci U S A. 2006; 103(8):2833-8. Epub 2006/02/13. Erratum in: Proc Natl Acad Sci U S A. 2006/12/26; 103(52):19931. https://doi: 10.1073/pnas.0511100103. PMID: 16477005.
7. Pearson JP, Pesci EC, Iglewski BH. Roles of *Pseudomonas aeruginosa* *las* and *rhl* quorum-sensing systems in control of elastase and rhamnolipid biosynthesis genes. J Bacteriol. 1997; 179(18):5756-67. https://doi: 10.1128/jb.179.18.5756-5767.1997. PMID: 9294432.
8. Kreiswirth BN, Löfdahl S, Betley MJ, O'Reilly M, Schlievert PM, Bergdoll MS, et al. The toxic shock syndrome exotoxin structural gene is not detectably transmitted by a prophage. Nature. 1983; 305(5936):709-12. https://doi: 10.1038/305709a0. PMID: 6226876.
9. Cheng Z, Zheng Y, Yang W, Sun H, Zhou F, Huang C, et al. Pathogenic bacteria exploit transferrin receptor transcytosis to penetrate the blood-brain barrier. Proc Natl Acad Sci U S A. 2023; 120(39):e2307899120. Epub 2023/09/21. https://doi: 10.1073/pnas.2307899120. PMID: 37733740.
