# Supplementary material for: Identification of the Pseudomonas aeruginosa AgtR-CspC-RsaL pathway that controls Las quorum sensing in response to metabolic perturbation and Staphylococcus aureus
Source: PLoS Pathog. 2025 Apr 8;21(4):e1013054. doi: 10.1371/journal.ppat.1013054 (PMC12051497; doi:10.1371/journal.ppat.1013054)
Supplement: S4 Table — (DOCX) [file ppat.1013054.s014.docx]

**S4 Table. Mass spectrometric result of the protein bands in the DNA pull-down assay.**

| **Gene ID-PA14** | **Gene ID-PAO1** | **Gene Name** | **MW[kDa]** | **Description** |
| --- | --- | --- | --- | --- |
| PA14_07840 | PA0601 | *agtR* | 22.9 | Putative two-component response regulator |
| PA14_44980 | PA1504 |  | 24.1 | Putative transcriptional regulator, TetR family |
| PA14_54510 | PA0756 |  | 24.4 | Probable two-component response regulator |
| PA14_25800 | PA2957 |  | 25 | Putative transcriptional regulator, TetR family |
| PA14_44690 | PA1526 |  | 25 | Putative transcriptional regulator, GntR family |
| PA14_03010 | PA0243 |  | 25.3 | Putative transcriptional regulator, TetR family |
| PA14_25180 | PA3006 | *psrA* | 25.8 | Transcriptional regulator PsrA |
| PA14_70560 | PA5344 |  | 34.3 | Putative transcriptional regulator, LysR family |
| PA14_41870 | PA1754 | *cysB* | 36.1 | Transcriptional regulator CysB |
| PA14_34660 | PA2320 | *gntR* | 37.1 | Transcriptional regulator GntR |
| PA14_35370 | PA2259 | *ptxS* | 37.4 | Transcriptional regulator PtxS |
| PA14_53520 | PA0831 | *oruR* | 37.6 | Transcriptional regulator OruR |
