# Supplementary material for: Identification of the Pseudomonas aeruginosa AgtR-CspC-RsaL pathway that controls Las quorum sensing in response to metabolic perturbation and Staphylococcus aureus
Source: PLoS Pathog. 2025 Apr 8;21(4):e1013054. doi: 10.1371/journal.ppat.1013054 (PMC12051497; doi:10.1371/journal.ppat.1013054)
Supplement: S1 Text — (DOCX) [file ppat.1013054.s015.docx]

**S1 Text. Supplemental Methods.**

Strains and plasmid construction.

Construction of the in-frame deletion mutants in *P. aeruginosa* was performed as described previously [1]. A donor strain S17-1 containing pEx18Tc-Δ*cspC* [2], a *cspC* gene deletion suicide plasmid, was used to construct the *tpiA cspC* dual-deletion mutant. The plasmid pEx18Tc-Δ*cspC* was transferred into the Δ*tpiA* mutant [3] by conjugation. The single-crossover mutants (strains with the plasmid integrated into the chromosome) were selected on LB plate with 100 μg/mL tetracycline and 25 μg/mL kanamycin to kill the S17-1 donor strain. The single-crossover mutants were cultured in LB overnight, and then plated on plates with 5% sucrose to select for double-crossover mutants. The correct deletion mutants were screened by PCR with primers delta-cspC-F and delta-cspC-R. For the deletion of *agtR*, a 986 bp and a 992 bp fragments upstream and downstream of the *agtR* coding region were amplified by PCR from the PA14 genome with primers agtR-U-F, agtR-U-R and agtR-D-F, agtR-D-R, respectively (S1 Table). These two fragments were mixed, followed by overlap PCR with primers agtR-U-F and agtR-D-R. Then the PCR product was cloned into the HindIII and BamHI sites of the plasmid pEX18Tc. Construction of single- and double-crossover mutants as well as screening for *agtR* deletion mutants were performed as aforementioned. For the deletion of the *phzA1B1C1D1E1F1G1* operon, a 960 bp and a 974 bp fragments upstream and downstream of the operon coding region were amplified by PCR from the PA14 genome with primers phz1-U-F, phz1-U-R and phz1-D-F, phz1-D-R, respectively (S1 Table). These two fragments were mixed, followed by overlap PCR with primers phz1-U-F and phz1-D-R. Then the PCR product was cloned into the HindIII and BamHI sites of the plasmid pEX18Tc. Construction of single- and double-crossover mutants as well as screening for *phzA1B1C1D1E1F1G1* operon deletion mutants were performed as aforementioned.

To construct the P*_rsaL_*-*lacZ* transcriptional fusion, a fragment containing 334 bp *rsaL* upstream region and 168 bp of *rsaL* coding region was amplified by PCR from the PA14 genome with primers EcoRI-PrsaL-F and BamHI-hrsaL-R (S1 Table). The PCR product was cloned into the EcoRI and BamHI sites of the plasmid pDN19lacΩ [4]. To construct the P*_cspC_-lacZ* transcriptional fusion, a fragment containing 439 bp *cspC* upstream region was amplified by PCR from the PA14 genome with primers EcoRI-PcspC-F and BamHI-PcspC-R (S1 Table). The PCR product was cloned into the EcoRI and BamHI sites of the plasmid pDN19lacΩ.

To construct the *rsaL*-*gst* translational fusion, the *rsaL* open reading frame with its promoter region was amplified by PCR from PA14 genome with primers EcoRI- PrsaL-F and PrsaL-rsaL-overlap-R (S1 Table). Another fragment was amplified by using pMMB67EH-74-*rsaL*-GST (S2 Table) plasmid as the template with primers rsaL-overlap-F and BamHI-GST-R (S1 Table). These two PCR products were mixed and then PCR was performed with primers EcoRI- PrsaL-F and BamHI-GST-R. The resultant PCR product was cloned into the EcoRI and BamHI sites of the promoterless plasmid pUCP20 [5], resulting in pUCP20-P*_rsaL_*-*rsaL-*GST.

To overexpress *agtR* and *gntR*, fragments containing the *agtR* and *gntR* coding regions were amplified by PCR from the PA14 genome with specific primers (S1 Table). The products were cloned into the EcoRI and BamHI sites of the plasmid pUCP20.

To construct the plasmid pET-His-SUMO-*agtR*, a fragment containing *agtR* coding region except the start codon was amplified by PCR from the PA14 genome with primers EcoRI-AgtR-F and HindIII-AgtR-R (S1 Table). The product was cloned into EcoRI and HindIII sites of the plasmid pET-His-SUMO.

To construct the mutP*_cspC_*-*lacZ* transcriptional fusion, a 303 bp and a 188 bp fragments were amplified by PCR using *cspC* promoter as a template with primers EcoRI-PcspC-F, mutPcspC-R and mutPcspC-F, BamHI-PcspC-R, respectively (S1 Table). These two fragments were mixed, followed by overlap PCR with primers EcoRI-PcspC-F and BamHI-PcspC-R. Then the PCR product was cloned into the EcoRI and BamHI sites of the plasmid pDN19lacΩ.

**References**

1. Hoang TT, Karkhoff-Schweizer RR, Kutchma AJ, Schweizer HP. A broad-host-range Flp-FRT recombination system for site-specific excision of chromosomally-located DNA sequences: application for isolation of unmarked *Pseudomonas aeruginosa* mutants. Gene. 1998; 212(1):77-86. https://doi: 10.1016/s0378-1119(98)00130-9. PMID: 9661666.
2. Li S, Weng Y, Li X, Yue Z, Chai Z, Zhang X, et al. Acetylation of the CspA family protein CspC controls the type III secretion system through translational regulation of *exsA* in *Pseudomonas aeruginosa*. Nucleic Acids Res. 2021; 49(12):6756-6770. https://doi: 10.1093/nar/gkab506. PMID: 34139014.
3. Xia Y, Wang D, Pan X, Xia B, Weng Y, Long Y, et al. TpiA is a key metabolic enzyme that affects virulence and resistance to aminoglycoside antibiotics through CrcZ in *Pseudomonas aeruginosa*. mBio. 2020; 11(1):e02079-19. https://doi: 10.1128/mBio.02079-19. PMID: 31911486.
4. Marx CJ, Lidstrom ME. Development of improved versatile broad-host-range vectors for use in methylotrophs and other Gram-negative bacteria. Microbiology (Reading). 2001; 147(Pt 8):2065-2075. https://doi: 10.1099/00221287-147-8-2065. PMID: 11495985.
5. Li M, Long Y, Liu Y, Liu Y, Chen R, Shi J, et al. HigB of *Pseudomonas aeruginosa* enhances killing of phagocytes by up-regulating the type III secretion system in ciprofloxacin induced persister cells. Front Cell Infect Microbiol. 2016; 6:125. https://doi: 10.3389/fcimb.2016.00125. PMID: 27790409.
