# Supplementary material for: Identification of the Pseudomonas aeruginosa AgtR-CspC-RsaL pathway that controls Las quorum sensing in response to metabolic perturbation and Staphylococcus aureus
Source: PLoS Pathog. 2025 Apr 8;21(4):e1013054. doi: 10.1371/journal.ppat.1013054 (PMC12051497; doi:10.1371/journal.ppat.1013054)
Supplement: Attachment — Submitted filename: Response to Reviewers.docx [file ppat.1013054.s017.docx]

Dear Editor and Reviewers:

Thank you for reviewing our manuscript entitled “Identification of the *Pseudomonas aeruginosa* AgtR-CspC-RsaL pathway that controls Las quorum sensing in response to metabolic perturbation and *Staphylococcus aureus*” (PPATHOGENS-D-24-02248). I appreciate the time and effort the reviewers have put into their comments. Those comments are very valuable and helpful for improving our manuscript. The following is a point-by-point synopsis of the changes we have made to address the comments and concerns.

**Part I – Summary**

Reviewer #1:

In this study, the authors demonstrated that the mutation of *tpiA* results in increased expression of QS genes, such as *lasI*, *rhlI*, and *pqsA*, as well as the production of QS signals. This upregulation is due to CspC mediated translational repression of *rsaL* in the *tpiA* mutant. The study also identifies AgtR as a regulator that upregulates *cspC* in the Δ*tpiA* mutant, and demonstrates that AgtR directly binds to the *cspC* promoter region to activate transcription. The authors also linked the AgtR-CspC pathway to the bacterial response to GlcNAc and competition with *Staphylococcus aureus,* providing a broader ecological context to their findings. The study concludes that the AgtR-CspC pathway plays a significant role in sensing environmental cues and in bacterial competition. Overall, the study is well-executed and provides valuable insights into the regulatory mechanisms of QS systems in *P. aeruginosa*.

**Response**: Thank you for your comment.

Reviewer #2:

In this research manuscript, Qu et al. present new data on the AgtR-CspC-RsaL-LasI pathway, and establish links between metabolism, quorum sensing (QS) and interspecies competition in *P. aeruginosa* PA14.

Appropriate experiments were conducted to investigate the subject and they appear well executed. Unfortunately, the main weakness of this paper is the writing, making for a frustrating read. It lacks important details in the introduction and the methodology, and a lot of references are inaccurate or misplaced. Also, the context of each result section and experiment is poorly introduced.

Also why look at all three QS system and not mention the implication of the rhl and PQS systems in the model? The authors focus on pyocyanin because it is overproduced in the *tpiA* mutant. But they also show induced expression of genes related to the las, rhl and PQS systems which together regulate a lot of factors which could also impact interaction with *Staphylococcus aureus*.

RsaL, which is mentioned in the paper to be regulated by CspC, is also a direct known regulator of the *phz* genes and thus pyocyanin production. Nothing is mentioned about this even if this would simply explain the enhanced pyocyanin production.

The pathway involved in sensing and competition against *S. aureus*: considering GlcNAc is a component of peptidoglycan, this response could be non-specific and a mechanism of defense against a wide variety of bacterium. It would be interesting to test the upregulation of this pathway against a variety of different bacterium and not only *S. aureus*.

The manuscript needs to be substantially rewritten for clarity and better context and with the right references throughout.

**Response**: We appreciate your comments. We have rewritten the manuscript to make it more clear. We also corrected some references that were inaccurate or misplaced.

As for QS systems, the Las system is at the top of the regulatory hierarchy, and our experiments were mainly conducted focused on Las system. As requested, we added the Rhl and PQS systems in Fig. 10. In addition, we added the description of the role of RsaL in regulating pyocyanin production.

As requested, we performed *P. aeruginosa* competition assay with *Streptococcus agalactiae* and *Staphylococcus epidermidis* (S10 Fig).

Reviewer #3:

This manuscript connects the regulators AgtR and CspC with phenotypes associated with deletions in the gene encoding TpiA, a central metabolism enzyme that converts between dihydroxyacetone phosphate and glycerol-3-phosphate (connecting glycolysis with glycerol metabolism). AgtR is a peptidoglycan-responsive regulator that has regulatory effects on several virulence factors, including quorum sensing. CspC is a recently described translation regulator that seems to have a number of different targets in the cell, several of which have roles in virulence. The studies show that some regulatory changes that occur in a ∆*tpiA* mutated strain occur through the AgtR-CspC pathway.

The results add to the current understanding of AgtR and CpsC regulatory functions and how these regulators connect with other networks in the cell. The results could be helpful for understanding changes in central metabolism or virulence regulation.

However, there were some major clarity and data interpretation issues in the manuscript that need addressed, as described below.

**Response:** Thank you for your comments.

**Part II – Major Issues: Key Experiments Required for Acceptance**

Reviewer #1:

1. The *phz1* and *phz2* operons share a high degree of homology. Which *phzA* mRNA level did the authors examine? I noticed that in the supplementary information, qRT-PCR primers for *phzB1* but not *phzA* were listed. Which gene was tested? The authors need to clarify whether the primers are operon-specific, or the overall *phzA* or *phzB* levels (e.g. *phzA1* + *phzA2*) were measured.

**Response**: Thank you for pointing out this mistake. The gene we tested is *phzA* (*phzA1* + *phzA2*). The primer sequences have been corrected in Table S1.

2. It seems AgtS-AgtR senses multiple ligands. Are there structural similarities between these ligands?

**Response**: Thank you for raising this question. A paper entitled “Functinal characterization of the *agtABCD* and *agtSR* operons for 4-aminobutyrate and 5-aminovalerate uptake and regulation in *Pseudomonas aeruginosa* PAO1.” reported that AgtS-AgtR can sense δ-aminovalerate, γ-aminobutyrate and β-alanine (Current Microbiology. 2014). Another paper entitled “Community surveillance enhances *Pseudomonas aeruginosa* virulence during polymicrobial infection.” reported that *N-*acetylglucosamine (GlcNAc) can be sensed by AgtS-AgtR (PNAS. 2013). All four compounds contain an amino group (-NH2). δ-Aminovalerate, γ-aminobutyrate, and β-alanine are amino acids with a carboxyl group (-COOH), while GlcNAc has an acetylamino group (-CONH2) instead of a free amino group. δ-aminovalerate, γ-aminobutyrate, and β-alanine contain a five-, four- and three-carbon chain, respectively. GlcNAc, on the other hand, is a larger molecule with an eight-carbon glucose backbone. Since all of the compounds are able to activate the AgtS-AgtR pathway, it is likely that they are ligands of AgtS. Further studies are warranted to examine the direct interaction between AgtS and each of the compounds. We added discussion in the Discussion section.

Reviewer #2:

1. When working on QS, it is important to consider timing of experimentations. The results obtained clearly show higher expression of QS-related genes. However, only one time point was chosen, precluding assessment of the QS effect. Does the *tipA* mutant grows similarly as the wildtype? Are results the same at other phases of growth? It would have been very useful to see this. The authors should at least demonstrate that the growth curve of Δ*tipA* is the same as that of the WT.

**Response**: Thank you for your question. We tested the expression of the QS genes the OD_600_ of 0.5. The expression of those genes were upregulated. The results were shown in S2 Figure. Besides, the growth curve of Δ*tipA* has been reported in the Figure S3 of our published paper entitled “TpiA is a key metabolic enzyme that affects virulence and resistance to aminoglycoside antibiotics through CrcZ in *Pseudomonas aeruginosa*. mBio. 2020”. The Δ*tipA* mutant grows more slowly than the wild type PA14. In our experiments we collected the bacteria when they reached the same growth phase (OD_600_). The description has been added to the main text (lines 130-141).

2. L139 : The title of the section named ‘’TpiA controls the QS systems through RsaL’’ is misleading. In this section, the authors indeed demonstrate that TpiA influences the transcription and translation of *rsaL*, a target of LasR, the key transcriptional regulator of the Las system. To clarify the direct effect of TpiA on the Las system, it would have been valuable to include data on the regulation of LasR itself. For example, assessing a reporter construct including *lasR* and its native promotor could verify whether TpiA directly impacts LasR, given the numerous regulators involved in controlling LasR expression. Perhaps a double mutant of *tpiA* and *rsaL* would have provided insight to look at the combined effect of these two genes on LasR regulation. Also, the impact on the other two QS systems is not described here.

**Response**: Thank you for pointing out the problem. We changed the section title to “TpiA controls the Las system through RsaL”. As requested, we tested the mRNA levels and the promoter activities of *lasR*. The transcriptional level of *lasR* was upregulated in the *tpiA* mutant (S4 Figure). Deletion of *rsaL* in wild type PA14 and the Δ*tipA* mutant increased the mRNA level of *lasI* to a similar level (S3 Figure), demonstrating that TpiA regulates *lasI* through RsaL. Deletion of *rsaL* in wild type PA14 increased the mRNA level of *lasR*. However, the expression levels of *lasR* were similar in the Δ*tipA* and Δ*tipA*Δ*rsaL* mutants (S3 Figure), indicating a different regulatory mechanism of *lasR* by RsaL. The description has been added to the manuscript (lines 167-174).

3. In the introduction, pyocyanin is mentioned as being important in the Pa and Sa interaction and the authors show that it is overexpressed in co-cultures in presence of Sa or with GlcNAc. This induction is lost in both the *agtR* and *cspC* mutants. This does not prove that pyocyanin is responsible for the competition observed. It could be another QS regulated factor. Comparing with a double *agtR phz* mutant, unable to produce pyocyanin would answer that question.

**Response**: Thank you for pointing out this problem. We performed the competition assay using the Δ*phz1* and Δ*agtR*Δ*phz1* mutants. The mutants displayed defective competition abilities against *S. aureus* (S9 Fig). The description has been added to the manuscript (lines 256-259).

Reviewer #3:

1. The interpretation of results with the *rsaL* transcription reporter is unclear. *rsaL* transcription is activated by LasR; if the LasR-I system is induced then *rsaL* will also be impacted. It seems likely the ∆*tpiA* mutation impacts *rsaL* transcription by modulating QS but it is not clear if this was the proposed mechanism.

It seems that the authors are proposing a mechanistic model whereby the ∆*tpiA* mutation leads to translational repression of RsaL, which leads to higher levels of QS. However, this model was never directly tested (e.g. by using an *rsaL* deletion or by examining QS activation in bacteria where RsaL regulation was unlinked from the ∆*tpiA* mutation).

Fig. 6C – it is unclear that the effects of the ∆*tpiA* and ∆*cspC* mutations are through the changes in RsaL levels. To test this directly, an *rsaL* deletion mutant would need to be tested, or by unlinking RsaL regulation from the ∆*tpiA* mutation).

**Response**: Thank you for your question. In our previous study, we reported that CspC represses the translation of *rsaL* (Acetylation of CspC controls the Las quorum-sensing system through translational regulation of *rsaL* in *Pseudomonas aeruginosa*. mBio. 2022). In the current study, our results demonstrated that although the transcription of *rsaL* was increased in the ∆*tpiA* mutant (Fig. 2A, B), the protein level of RsaL was reduced (Fig. 2C). By using a *tac* promoter driven *rsaL*, we demonstrated that the translation of *rsaL* was reduced in the ∆*tpiA* mutant (Fig. 3).

As requested, we verify the role of RsaL by using a ∆*rsaL* mutant and a ∆*tpiA*∆*rsaL* double mutant. Deletion of *rsaL* increased the expression of *lasI* (S3 Fig). Description of the results has been added to the manuscript (lines 167-174).

2. Fig. 10: what is RN4220? There is no mention of this in the legend or methods, unless something was missed.

**Response**: Thank you for your question. RN4220 is a widely used *S. aureus* strain. We have added the description of RN4220 in the legend of Fig. 9 (Fig 10 in the previously submitted version). It is also described in the S2 Table.

**Part III – Minor Issues: Editorial and Data Presentation Modifications**

Reviewer #1:

1. “illustated” should be “illustrated” in the discussion section.

**Response**: Thank you for catching this mistake. We have corrected it.

2. The language used in the manuscript is generally clear, but some sentences are quite dense and could be broken down for better readability, e.g. line 256-258.

**Response**: Thank you for bringing this to our attention. We have rewritten the sentence in line 280-282 (line 256-258 in the previously submitted version).

3. Fig 1, 2, 3, 5, the authors should clarify which samples were compared.

**Response**: Thank you for your suggestion. We have indicated the objects which samples were compared in Fig 1, 2, 3, 5.

4. Line 269, what does “coenzyme” mean here?

**Response**: It is a mistake. The word has been deleted.

5. Line 270, it is better to replace “agtA” with “the agtABCD operon”.

**Response**: We appreciate your suggestion. We have replaced “*agtA*” with “the *agtABCD* operon”.

Reviewer #2:

1. Correct the following molecules throughout the text so that the number of atoms are in subscript: 3-oxo-C12-HSL; C4-HSL

2. Line 48: Demonstrate

3. Line 53: co-exists

**Response to 1-3**: Thank you for pointing out the mistakes. We have made the corrections accordingly.

4. Line 70, A better reference for this should be Mavrodi et al. J. Bacteriol. 2001; 183(21) : 6454-65

**Response**: Thank you for your suggestion. We have replaced the reference.

5. Line 71, the wrong reference is cited. A suggestion would be Latifi et al. Mol Microbiol. 1995;17(2)333-43

**Response**: Thank you for your suggestion. We have replaced the reference.

6. Ln 78-81: This sentence is not clear. PqsH is implicated only in the synthesis of PQS and it seems the sentence was written as if it were HHQ. Also, the chosen references are not appropriate for describing this system.

**Response**: Thank you for pointing out the mistake. We have rewritten the sentence as “The PQS system includes the regulatory protein PqsR and two signaling molecules, 2-heptyl-3-hydroxy-4-quinolone (PQS) and 2-heptyl-4-quinolone (HHQ), synthesized by proteins encoded by the *pqsH* gene, the *pqsABCDE*, and *phnAB* operons.” The references have been replaced with the more appropriate ones.

7. Line 81: Again, these references are not appropriate.

**Response**: We have replaced the references with the more appropriate ones.

8. Line 84 Suppresses is a strong word. Maybe inhibit?

**Response**: Thank you for your suggestion. We have replaced “suppresses” with “inhibits”.

9. Line 85: Again, wrong reference.

**Response**: Thank you for pointing out the mistake. We have replaced the reference with a more appropriate one.

10. Line 126 and others: it should be RT-qPCR instead of qRT-PCR.

**Response**: We have replaced all “qRT-PCR” with “RT-qPCR”.

11. Line 142: Again, this is not the best reference for this claim.

**Response**: Thank you for your suggestion. We have replaced the reference with a more appropriate one.

12. Figure 2C: Description is lacking in the legend. Miller Units does not say anything on what was used to measure the signals. We should not have to go to the material and method to understand what we see. Moreover, this section is not well described in material and method. Firstly, once again, the reference cited has nothing to do with what is described and we need to refer to Supplementary Tables to try and understand which tool was used.

**Response**: We apologize for the confusion and mistake. We have added description in the legend of Figure 1C (Fig 2C in the previously submitted manuscript). More detailed description has also been added to the Method section named “QS signal molecular reporter assay”. The reference has been replaced with correct ones.

13. Figure 2C: it is likely your reporter detects HHQ as well as PQS and what you measure is a combination of both. Know the limits of the methods used.

**Response**: Thank you for pointing out the issue. We revised the description in the figure legend and manuscript.

14. Line 156: where are the references for those previous studies?

**Response**: We added the reference at the end of the sentence.

15. You first mention the role of AgtR at line 230. It would have been important to include a more through description of this regulator and its context in the introduction, as it is only briefly mentioned at line 93. Also, maybe include more information as to why you chose AgtR to pursue your experiments, and not GntR or PA0756, who are also upregulated in a tpiA mutant.

**Response**: Thank you for your inquiry. We added the description of AgtR in the introduction section (lines 94-97).

In the experiments to identify the *cspC*’s regulator, we examined the expression of *cspC* in stains with mutations or overexpressing the candidate genes. Only AgtR affected *cspC* expression under both scenarios, i.e., mutation of *agtR* reduced the expression of *cspC*, while overexpression of *agtR* increased the expression of *cspC* (S6 Fig). We revised the sentences for clarification (lines 206-214).

16. Fig 7B: it is hard to distinguish between the different levels of greys for each strain.

**Response**: We apologize for the lack of clarity in the figure. We have adjusted the greys of each strain in Fig 6 (Fig. 7 in the previously submitted version).

17. Line 306: what statistical method was used for qPCR? Was it 2ΔΔCT? Is rspL known to be a good internal control? Is there a reference? If not, authors should show the levels of expression in the different backgrounds to demonstrate it is similar.

**Response**: We used the statistical method of 2^ΔΔCT^ for qPCR. *rpsL* has been used as the internal control in multiple studies. We added the references and description of the statistical method in the method section.

18. Line 333: should still be a better description of the tool used to quantity PQS than Pseudomonas reporter strain.

**Response**: We added more detail in the method section “QS signal molecular reporter assay” and added the information of the reporter in Table S2.

19. Line 454: ratio and (add space between the two words)

**Response**: Thank you for catching this mistake. We have corrected it.

20. Fig 10: If you use RN4220 in the figure, it should be described in the legend.

**Response**: We have added the description of RN4220 in the legend of Fig 9 (Fig 10 in the previously submitted version).

21. Table S2: details should be added on strains listed as Laboratory stocks. Were these previously published? If not the details on their construction should be included. Were they received from another lab? The reference should be added.

**Response**: Thank you for pointing out this issue. We have updated the Table S2 as required. The references have been added.

22. Competition assay: it is not clear how were chosen the ratio for competition and what is the impact of this ratio on the results.

**Response**: The *P. aeruginosa*-*S. aureus* competition assay was performed as previously describe with minor modifications. The reference has been added to the manuscript. We changed the initial *P. aeruginosa*: *S. aureus* ratio from 1:9 (reference #8 and #59) to 1:30, which displayed greater difference between wild type PA14 and the tested mutants. As for *S. agalactiae* and *S. epidermidis*, no reference was found. We conducted preliminary experiments to determine the growth rates of the strains and chose the ratios that resulted in obvious differences between the *P. aeruginosa* strains.

Reviewer #3:

1. Lines 71-74: It is not clear what is meant by QS enabling responses to other environmental signals or species composition. Consider rewording the description of QS to “a bacterial cell-cell communication system that enables cells to respond to changes in cell density” as this is less confusing.

**Response**: Thank you for your kind suggestion. We have reworded the sentence accordingly (lines 71-72).

2. Figures 1 and 2 can be combined, or Fig. 1 could be moved to the supplement.

**Response**: Thank you for your suggestion. We have moved Fig. 1 to the supplement as S1 Fig.

3. Lines 194-195: it is unclear what is meant by, “among which the mRNA levels of gntR, PA0756 and *agtR* were elevated in the ΔtpiA mutant (S2B Fig).” It seems from the figure that the mRNA levels were measured for each of these genes in the ∆tpiA mutant but the way this sentence is written it sounds like the mRNA was somehow measured during the biotin pulldown experiment.

**Response**: We apologize for the confusion. We have reworded the sentence as “We then determined the expression levels of those genes by RT-qPCR. The mRNA levels of *gntR*, *PA0756* and *agtR* were elevated in the Δ*tpiA* mutant (S6B Fig).” (lines 206-207)

4. Line 224: the text needs to modified to clarify that lacZ is measuring cpsC expression levels

**Response**: Thank you for your suggestion. We have rewritten the sentence as “mutating the binding sequence in the P*_cspC_*-*lacZ* transcriptional fusion reduced the *lacZ* expression in both the Δ*tpiA* mutant and the *agtR* overexpression strain to wild type levels (Fig 8D), demonstrating reduction of the *cspC* promoter activity.” (lines 236-239)

Oher changes:

“LB” shown in Fig. 9A and Fig. 9G was corrected as “M9”.

Again, we would like to extend our gratitude to the reviewers for bringing these issues to our attention. My coauthors and I agree that the changes made to the manuscript in addressing the reviewers’ concerns have greatly improved this manuscript.

Best regards,

Weihui Wu
