# Supplementary material for: Identification of the Pseudomonas aeruginosa AgtR-CspC-RsaL pathway that controls Las quorum sensing in response to metabolic perturbation and Staphylococcus aureus
Source: PLoS Pathog. 2025 Apr 8;21(4):e1013054. doi: 10.1371/journal.ppat.1013054 (PMC12051497; doi:10.1371/journal.ppat.1013054)
Supplement: Attachment — Submitted filename: Response_to_Reviewers_auresp_2.docx [file ppat.1013054.s018.docx]

Dear Editors and Reviewers:

Thank you for reviewing our manuscript. We are very grateful for the constructive comment. The following is our response to the comment.

Reviewer #2: I am mostly satisfied with the revised version.

However, I still have one concern that must be addressed. About my question on any growth rate difference between the tpiA mutant and the WT, which might influence the quorum sensing response, the authors confirm that the mutant is growing slower and refer me to the supplementary data of a previously published paper. I am not fully satisfied by this answer. I checked, and the growth defect is actually important. It must be clearly mentioned in the present manuscript that the mutant has a growth defect. The authors state that they matched cultures by OD600 instead of timing, this likely represents several hours of difference in timing of sampling. While I agree that this could be OK when looking at gene expression, the same does not goes for measurement of accumulated metabolites, for instance. The authors should simply mention that because of the growth defect, cultures we sampled at corresponding OD600 (the growth curves in the previous paper does allow to verify if this corresponds to the same growth stage, so the authors cannot claim that, as we do not know if the mutant can eventually reach the same maximal cell density…In fact, according to the growth curves shown in the previous paper, it is very likely that it never reaches the same OD600 as the WT). The authors should keep in mind that OD600 measurements are only a proxy, and the actual information must be obtained by counting CFUs, anyway.

**Response**: Thank you for pointing out the issue. We cultured the bacteria with the same initial concentration and harvested the cultures at the same OD_600_. In our experiments, we determined the concentrations of PA14, the Δ*tpiA* mutant and the complemented strain at the sampling OD_600_ (0.5 and 2.5). Their concentrations (CFU/mL) were almost the same. The culture time and concentrations of each strain were listed in S1 Table. As requested, we added the description of the growth defect of the Δ*tpiA* mutant and different sample collection time in the manuscript (line 128-133).

Again, we would like to extend our gratitude to the reviewers for bringing the issue to our attention. Please contact me if I can provide any additional information.

Best regards,

Wu Weihui
