# Supplementary figures and images for: Correction: Identification of the Pseudomonas aeruginosa AgtR-CspC-RsaL pathway that controls Las quorum sensing in response to metabolic perturbation and Staphylococcus aureus
Source: PLoS Pathog. 2026 Feb 10;22(2):e1013944. doi: 10.1371/journal.ppat.1013944 (PMC12890109; doi:10.1371/journal.ppat.1013944)

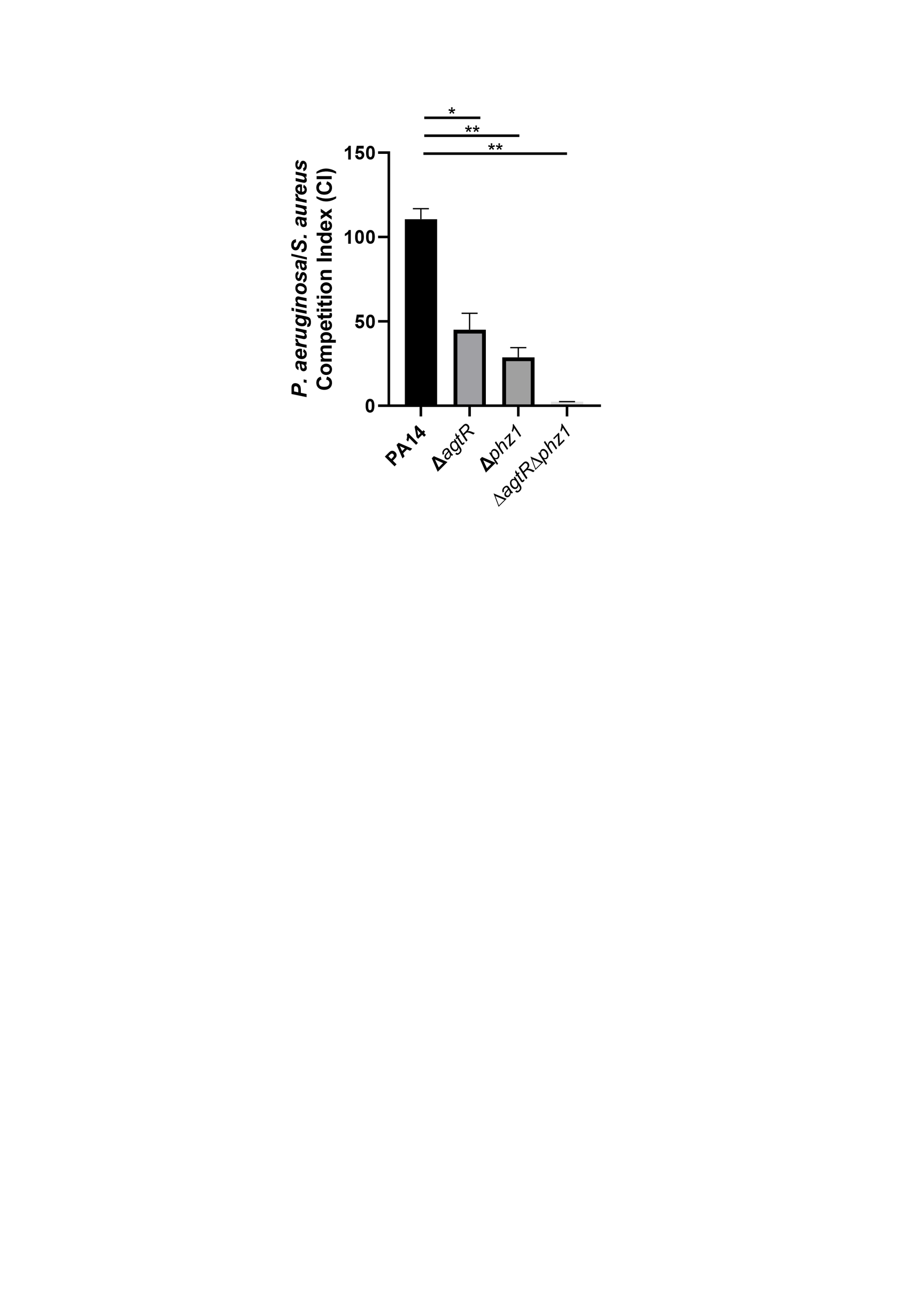

Supplement: S9 Fig — Bacteria were grown overnight in LB at 37 °C. Indicated P. aeruginosa strains and S. aureus RN4220 were mixed at a 1:30 ratio and cocultured in LB at 37 °C for 14 h. The competitive indexes represent the ratio of P. aeruginosa to S. aureus. Data represent the mean ± standard deviation of the results from three samples. **, P < 0.01; *, P < 0.05 by Student’s t test. (TIF) [file ppat.1013944.s001.tif]

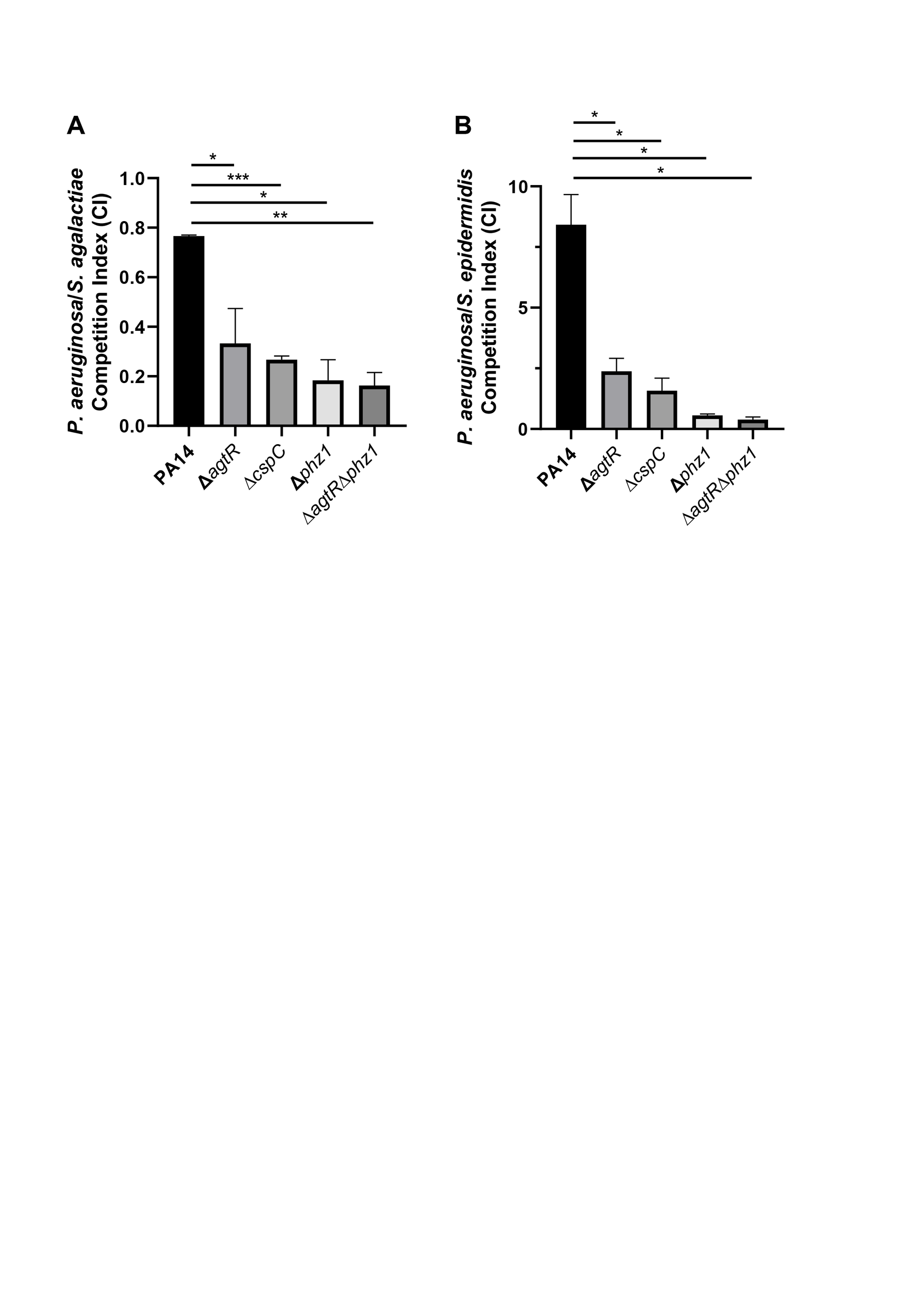

Supplement: S10 Fig — Bacteria were grown overnight in LB at 37 °C. (A) P. aeruginosa and S. agalactiae were mixed at a 1:3 ratio and cocultured in LB at 37 °C for 6 h. The competitive indexes represent the ratio of P. aeruginosa to S. agalactiae. (B) P. aeruginosa and S. epidermidis were mixed at a 1:30 ratio and cocultured in LB at 37 °C for 6 h. The competitive indexes represent the ratio of P. aeruginosa to S. epidermidis. Data represent the mean ± standard deviation of the results from three samples. ***, P < 0.001; **, P < 0.01; *, P < 0.05 by Student’s t test. (TIF) [file ppat.1013944.s002.tif]
